# *N*,*N*′-Bis(2-chloro­phen­yl)propane­diamide

**DOI:** 10.1107/S1600536810044090

**Published:** 2010-10-31

**Authors:** B. Thimme Gowda, Miroslav Tokarčík, Vinola Z. Rodrigues, Jozef Kožíšek, Hartmut Fuess

**Affiliations:** a Department of Chemistry, Mangalore University, Mangalagangotri 574 199, Mangalore, India; b Faculty of Chemical and Food Technology, Slovak Technical University, Radlinského 9, SK-812 37 Bratislava, Slovak Republic; c Institute of Materials Science, Darmstadt University of Technology, Petersenstrasse 23, D-64287 Darmstadt, Germany

## Abstract

The crystal structure of the title compound, C_15_H_12_Cl_2_N_2_O_2_, contains three intramolecular hydrogen bonds; two C—H⋯O and a nonclassical N—H⋯Cl. The structure is further stabilized by intermolecular N—H⋯O hydrogen bonds and C—H⋯π interactions, resulting in a three-dimensional network. The two benzene rings make an interplanar angle of 58.0 (1)°.

## Related literature

For literature on related compounds, see: Gowda *et al.* (2007 ▶, 2009 ▶, 2010 ▶).
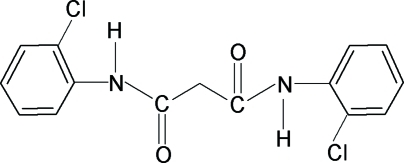


## Experimental

### 

#### Crystal data

C_15_H_12_Cl_2_N_2_O_2_
*M* *_r_* = 323.17
Monoclinic, 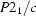

*a* = 13.8819 (9) Å
*b* = 15.3556 (10) Å
*c* = 7.0316 (5) Å
β = 104.027 (7)°
*V* = 1454.19 (17) Å^3^
*Z* = 4
Mo *K*α radiation
μ = 0.45 mm^−1^
*T* = 295 K
0.57 × 0.54 × 0.15 mm

#### Data collection

Oxford Diffraction Gemini R CCD diffractometer
Absorption correction: analytical (*CrysAlis PRO*; Oxford Diffraction, 2009 ▶) *T*_min_ = 0.743, *T*_max_ = 0.938
13088 measured reflections
2687 independent reflections
1930 reflections with *I* > 2σ(*I*)
*R*_int_ = 0.042

#### Refinement

*R*[*F* ^2^ > 2σ(*F* ^2^)] = 0.044
*wR*(*F* ^2^) = 0.127
*S* = 1.03
2687 reflections
190 parameters
H-atom parameters constrained
Δρ_max_ = 0.19 e Å^−3^
Δρ_min_ = −0.39 e Å^−3^

### 

Data collection: *CrysAlis PRO* (Oxford Diffraction, 2009 ▶); cell refinement: *CrysAlis PRO*; data reduction: *CrysAlis PRO*; program(s) used to solve structure: *SHELXS97* (Sheldrick, 2008 ▶); program(s) used to refine structure: *SHELXL97* (Sheldrick, 2008 ▶); molecular graphics: *ORTEP-3* (Farrugia, 1997 ▶) and *DIAMOND* (Brandenburg, 2002 ▶); software used to prepare material for publication: *SHELXL97*, *PLATON* (Spek, 2009 ▶) and *WinGX* (Farrugia, 1999 ▶).

## Supplementary Material

Crystal structure: contains datablocks I, global. DOI: 10.1107/S1600536810044090/bt5396sup1.cif

Structure factors: contains datablocks I. DOI: 10.1107/S1600536810044090/bt5396Isup2.hkl

Additional supplementary materials:  crystallographic information; 3D view; checkCIF report

## Figures and Tables

**Table 1:** Hydrogen-bond geometry (Å, °) *Cg*2 is the centroid of the C10/C15 phenyl ring.

*D*—H⋯*A*	*D*—H	H⋯*A*	*D*⋯*A*	*D*—H⋯*A*
N1—H1N⋯O2^i^	0.86	2.24	3.038 (2)	154
N2—H2N⋯O1^ii^	0.86	2.03	2.856 (2)	160
C8—H8*A*⋯O2^i^	0.97	2.43	3.219 (3)	138
C3—H3⋯*Cg*2^iv^	0.93	2.74	3.608 (2)	155
C15—H15⋯O2	0.93	2.52	2.916 (3)	106
N1—H1N⋯Cl1	0.86	2.58	2.9730 (18)	109

Symmetry codes: (i) 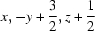; (ii) 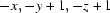; (iii) 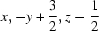; (iv) 
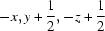
.

**Fig. 1.**
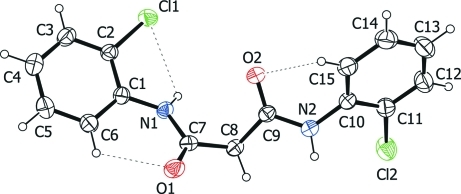
Molecular structure of (I) showing the atom labelling scheme. Displacement ellipsoids are drawn at the 30% probability level. H atoms are represented as small spheres of arbitrary radii. Three intramolecular hydrogen bonds are shown as dashed lines.

**Fig. 2.**
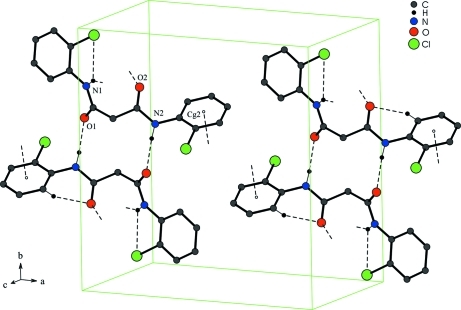
Packing diagram of (I) with hydrogen bonds indicated by dashed lines. The hydrogen atoms not participating in hydrogen bonding have been omitted. Cg2 is the centroid of the C10/C15 phenyl ring.

## supplementary crystallographic information

### Comment

The amide moiety is an important constituent of many biologically significant compounds. As a part of studying the effect of substitutions on the structures of this class of compounds (Gowda *et al.*, 2007; 2009; 2010), the crystal structure of *N*,*N*-bis(2-chlorophenyl)-malonamide has been determined (I) (Fig. 1).

The molecular structure of (I) includes three intermolecular hydrogen bonds (Table 1); two of them are C–H···O hydrogen bonds, the third is a non- classical N–H···Cl hydrogen bond. The two phenyl rings make an interplanar angle of 58.0 (1)°. The dihedral angle made by the two amido groups is 65.0 (2)°. The conformation of the *ortho*-chlorosubstituent is *anti* to the nearest carbonyl C=O bond, as indicated by the torsion angles, C2—C1—N1—C7 = -156.1 (2)° and C11—C10—N2—C9 = 137.2 (2)° in the first and the second phenyl rings, respectively. The chlorine Cl atom attached to the C1/C6 phenyl ring gives rise to a non conventional N–H···Cl hydrogen bond, with N–Cl distance of 2.9730 (18) Å and angle of 109°. The second chlorine atom, attached to the C10/C15 phenyl ring, makes a short intramolecular contact of 2.960 (2)Å with the nearest amide N atom, forming the N–H···Cl angle of 98°. In the crystal, the molecules are linked by intermolecular N–H···O hydrogen bonds into the chains running along the base vector [0 1 1] parallel to the *bc*-plane (Fig. 2). The chains are further stabilized by C–H···π interaction between the C3 atom of the C1/C6 ring and the centroid *Cg*2 of the phenyl ring C10/C15 at the position (-*x*, *y* + 1/2, -*z* + 1/2).

### Experimental

Malonic acid (0.3 mol) in dichloromethane (30 ml) was treated with 2-chloroaniline (0.6 mol) in dichloromethane (30 ml), dropwise with stirring. The resulting mixture was stirred for 3 hrs and kept aside for 12 hrs for the completion of reaction and evaporation of the solvent, dichloromethane. The product obtained was added to crushed ice to obtain the precipitate. The latter was thoroughly washed with water and then with saturated sodium bicarbonate solution and washed again with water. It was then given a wash with 2 N HCl. It was again washed with water, filtered, dried and recrystallized to the constant melting point from ethanol.

Block like colorless single crystals of the title compound used in X-ray diffraction studies were obtained by a slow evaporation of its ehanolic solution at room temperature.

### Refinement

All H atoms were positioned geometrically and refined using a riding model with C–H = 0.93 or 0.97 Å and N–H = 0.86 Å. The *U*_iso_(H) values were set at 1.2*U*_eq_(C, N).

### Crystal data

**Table:**

C_15_H_12_Cl_2_N_2_O_2_	*F*(000) = 664
*M**_r_* = 323.17	*D*_x_ = 1.476 Mg m^−^^3^
Monoclinic, *P*2_1_/*c*	Mo *K*α radiation, λ = 0.71073 Å
Hall symbol: -P 2ybc	Cell parameters from 6793 reflections
*a* = 13.8819 (9) Å	θ = 3.7–29.3°
*b* = 15.3556 (10) Å	µ = 0.45 mm^−^^1^
*c* = 7.0316 (5) Å	*T* = 295 K
β = 104.027 (7)°	Block, colorless
*V* = 1454.19 (17) Å^3^	0.57 × 0.54 × 0.14 mm
*Z* = 4	

### Data collection

**Table:**

Oxford Diffraction Gemini R CCD diffractometer	2687 independent reflections
graphite	1930 reflections with *I* > 2σ(*I*)
Detector resolution: 10.434 pixels mm^-1^	*R*_int_ = 0.042
ω scans	θ_max_ = 25.4°, θ_min_ = 2.7°
Absorption correction: analytical (*CrysAlis PRO*; Oxford Diffraction, 2009)	*h* = −15→16
*T*_min_ = 0.743, *T*_max_ = 0.938	*k* = −15→18
13088 measured reflections	*l* = −8→8

### Refinement

**Table:**

Refinement on *F*^2^	Primary atom site location: structure-invariant direct methods
Least-squares matrix: full	Secondary atom site location: difference Fourier map
*R*[*F*^2^ > 2σ(*F*^2^)] = 0.044	Hydrogen site location: inferred from neighbouring sites
*wR*(*F*^2^) = 0.127	H-atom parameters constrained
*S* = 1.03	*w* = 1/[σ^2^(*F*_o_^2^) + (0.0805*P*)^2^] where *P* = (*F*_o_^2^ + 2*F*_c_^2^)/3
2687 reflections	(Δ/σ)_max_ = 0.001
190 parameters	Δρ_max_ = 0.19 e Å^−^^3^
0 restraints	Δρ_min_ = −0.39 e Å^−^^3^

### Special details

**Table:**

Geometry. All e.s.d.'s (except the e.s.d. in the dihedral angle between two l.s. planes) are estimated using the full covariance matrix. The cell e.s.d.'s are taken into account individually in the estimation of e.s.d.'s in distances, angles and torsion angles; correlations between e.s.d.'s in cell parameters are only used when they are defined by crystal symmetry. An approximate (isotropic) treatment of cell e.s.d.'s is used for estimating e.s.d.'s involving l.s. planes.
Refinement. Refinement of *F*^2^ against ALL reflections. The weighted *R*-factor *wR* and goodness of fit *S* are based on *F*^2^, conventional *R*-factors *R* are based on *F*, with *F* set to zero for negative *F*^2^. The threshold expression of *F*^2^ > σ(*F*^2^) is used only for calculating *R*-factors(gt) *etc*. and is not relevant to the choice of reflections for refinement. *R*-factors based on *F*^2^ are statistically about twice as large as those based on *F*, and *R*- factors based on ALL data will be even larger.

### Fractional atomic coordinates and isotropic or equivalent isotropic displacement parameters (Å^2^)

**Table:**

	*x*	*y*	*z*	*U*_iso_*/*U*_eq_	
C1	−0.19293 (15)	0.77176 (14)	0.5062 (3)	0.0449 (5)	
C2	−0.19188 (15)	0.86196 (14)	0.4842 (3)	0.0469 (5)	
C3	−0.27859 (19)	0.90983 (16)	0.4347 (3)	0.0582 (6)	
H3	−0.2764	0.97	0.422	0.07*	
C4	−0.36823 (18)	0.86717 (19)	0.4044 (4)	0.0666 (7)	
H4	−0.4272	0.8986	0.3704	0.08*	
C5	−0.37089 (17)	0.77888 (18)	0.4241 (4)	0.0635 (7)	
H5	−0.4319	0.7508	0.4031	0.076*	
C6	−0.28444 (16)	0.73034 (16)	0.4747 (3)	0.0543 (6)	
H6	−0.2876	0.6702	0.4875	0.065*	
C7	−0.08275 (16)	0.64231 (14)	0.5353 (3)	0.0444 (5)	
C8	0.02513 (16)	0.61601 (14)	0.6081 (3)	0.0465 (5)	
H8A	0.0515	0.6411	0.7369	0.056*	
H8B	0.0292	0.5531	0.6211	0.056*	
C9	0.08797 (15)	0.64552 (15)	0.4718 (3)	0.0437 (5)	
C10	0.23819 (17)	0.60854 (14)	0.3636 (3)	0.0494 (5)	
C11	0.33523 (18)	0.58501 (16)	0.4524 (3)	0.0567 (6)	
C12	0.4116 (2)	0.60164 (18)	0.3623 (4)	0.0701 (7)	
H12	0.4763	0.585	0.4224	0.084*	
C13	0.3912 (2)	0.6429 (2)	0.1836 (4)	0.0764 (8)	
H13	0.4422	0.6547	0.1228	0.092*	
C14	0.2958 (2)	0.66657 (18)	0.0949 (4)	0.0710 (7)	
H14	0.2825	0.6947	−0.0258	0.085*	
C15	0.21891 (19)	0.64911 (16)	0.1830 (4)	0.0595 (6)	
H15	0.1542	0.6647	0.1206	0.071*	
N1	−0.10196 (12)	0.72631 (11)	0.5652 (3)	0.0479 (4)	
H1N	−0.0523	0.7564	0.6287	0.057*	
N2	0.16193 (13)	0.59006 (12)	0.4599 (3)	0.0514 (5)	
H2N	0.1627	0.5399	0.5148	0.062*	
O1	−0.14642 (13)	0.59039 (10)	0.4540 (3)	0.0607 (4)	
O2	0.07446 (11)	0.71541 (10)	0.3868 (2)	0.0543 (4)	
Cl1	−0.07891 (4)	0.91714 (4)	0.52012 (9)	0.0599 (2)	
Cl2	0.36153 (5)	0.53220 (6)	0.67829 (10)	0.0792 (3)	

### Atomic displacement parameters (Å^2^)

**Table:**

	*U*^11^	*U*^22^	*U*^33^	*U*^12^	*U*^13^	*U*^23^
C1	0.0449 (12)	0.0454 (14)	0.0446 (11)	0.0017 (9)	0.0115 (9)	−0.0026 (9)
C2	0.0521 (13)	0.0417 (14)	0.0490 (12)	0.0019 (10)	0.0164 (10)	−0.0002 (9)
C3	0.0653 (16)	0.0489 (15)	0.0625 (15)	0.0124 (12)	0.0192 (12)	0.0028 (11)
C4	0.0545 (15)	0.072 (2)	0.0730 (17)	0.0135 (13)	0.0144 (12)	0.0016 (13)
C5	0.0466 (13)	0.074 (2)	0.0698 (16)	−0.0023 (12)	0.0144 (11)	−0.0043 (13)
C6	0.0515 (13)	0.0530 (15)	0.0605 (13)	−0.0026 (11)	0.0173 (11)	−0.0016 (11)
C7	0.0541 (13)	0.0376 (13)	0.0424 (11)	−0.0014 (10)	0.0134 (10)	0.0026 (9)
C8	0.0534 (13)	0.0413 (13)	0.0449 (11)	0.0057 (10)	0.0121 (10)	0.0009 (9)
C9	0.0464 (12)	0.0392 (13)	0.0438 (11)	−0.0002 (10)	0.0075 (9)	−0.0033 (9)
C10	0.0526 (13)	0.0401 (13)	0.0584 (13)	0.0017 (10)	0.0189 (10)	−0.0028 (10)
C11	0.0546 (14)	0.0564 (16)	0.0598 (14)	−0.0020 (11)	0.0151 (11)	−0.0047 (11)
C12	0.0548 (15)	0.077 (2)	0.0827 (19)	−0.0055 (13)	0.0240 (14)	−0.0054 (15)
C13	0.0774 (19)	0.077 (2)	0.088 (2)	−0.0130 (16)	0.0453 (16)	−0.0037 (16)
C14	0.091 (2)	0.0587 (17)	0.0704 (16)	−0.0004 (14)	0.0334 (15)	0.0047 (13)
C15	0.0691 (16)	0.0501 (14)	0.0620 (14)	0.0044 (12)	0.0210 (12)	0.0043 (11)
N1	0.0429 (10)	0.0368 (11)	0.0623 (11)	−0.0002 (8)	0.0096 (8)	−0.0050 (8)
N2	0.0529 (11)	0.0420 (11)	0.0614 (11)	0.0048 (8)	0.0178 (9)	0.0070 (8)
O1	0.0604 (10)	0.0435 (10)	0.0734 (11)	−0.0032 (8)	0.0069 (8)	−0.0059 (8)
O2	0.0569 (9)	0.0424 (10)	0.0649 (9)	0.0056 (7)	0.0172 (7)	0.0097 (7)
Cl1	0.0637 (4)	0.0444 (4)	0.0747 (4)	−0.0066 (3)	0.0230 (3)	−0.0025 (3)
Cl2	0.0572 (4)	0.1069 (6)	0.0703 (5)	0.0077 (3)	0.0092 (3)	0.0168 (4)

### Geometric parameters (Å, °)

**Table:**

C1—C6	1.390 (3)	C8—H8B	0.97
C1—C2	1.394 (3)	C9—O2	1.221 (3)
C1—N1	1.414 (3)	C9—N2	1.352 (3)
C2—C3	1.381 (3)	C10—C15	1.381 (3)
C2—Cl1	1.746 (2)	C10—C11	1.388 (3)
C3—C4	1.376 (3)	C10—N2	1.417 (3)
C3—H3	0.93	C11—C12	1.385 (3)
C4—C5	1.364 (4)	C11—Cl2	1.742 (2)
C4—H4	0.93	C12—C13	1.374 (4)
C5—C6	1.384 (3)	C12—H12	0.93
C5—H5	0.93	C13—C14	1.370 (4)
C6—H6	0.93	C13—H13	0.93
C7—O1	1.224 (2)	C14—C15	1.384 (3)
C7—N1	1.344 (3)	C14—H14	0.93
C7—C8	1.515 (3)	C15—H15	0.93
C8—C9	1.514 (3)	N1—H1N	0.86
C8—H8A	0.97	N2—H2N	0.86
			
C6—C1—C2	118.09 (19)	O2—C9—N2	123.54 (19)
C6—C1—N1	122.5 (2)	O2—C9—C8	121.98 (18)
C2—C1—N1	119.36 (18)	N2—C9—C8	114.42 (19)
C3—C2—C1	121.7 (2)	C15—C10—C11	118.8 (2)
C3—C2—Cl1	118.39 (18)	C15—C10—N2	121.9 (2)
C1—C2—Cl1	119.95 (16)	C11—C10—N2	119.3 (2)
C4—C3—C2	119.1 (2)	C12—C11—C10	120.9 (2)
C4—C3—H3	120.5	C12—C11—Cl2	119.2 (2)
C2—C3—H3	120.5	C10—C11—Cl2	119.83 (18)
C5—C4—C3	120.2 (2)	C13—C12—C11	119.5 (3)
C5—C4—H4	119.9	C13—C12—H12	120.3
C3—C4—H4	119.9	C11—C12—H12	120.3
C4—C5—C6	121.2 (2)	C14—C13—C12	120.1 (3)
C4—C5—H5	119.4	C14—C13—H13	119.9
C6—C5—H5	119.4	C12—C13—H13	119.9
C5—C6—C1	119.8 (2)	C13—C14—C15	120.7 (3)
C5—C6—H6	120.1	C13—C14—H14	119.7
C1—C6—H6	120.1	C15—C14—H14	119.7
O1—C7—N1	123.4 (2)	C10—C15—C14	120.0 (2)
O1—C7—C8	121.79 (19)	C10—C15—H15	120
N1—C7—C8	114.84 (19)	C14—C15—H15	120
C9—C8—C7	112.33 (17)	C7—N1—C1	128.66 (18)
C9—C8—H8A	109.1	C7—N1—H1N	115.7
C7—C8—H8A	109.1	C1—N1—H1N	115.7
C9—C8—H8B	109.1	C9—N2—C10	124.75 (19)
C7—C8—H8B	109.1	C9—N2—H2N	117.6
H8A—C8—H8B	107.9	C10—N2—H2N	117.6
			
C6—C1—C2—C3	0.7 (3)	C15—C10—C11—Cl2	−178.97 (18)
N1—C1—C2—C3	−177.32 (19)	N2—C10—C11—Cl2	0.8 (3)
C6—C1—C2—Cl1	−179.48 (15)	C10—C11—C12—C13	0.7 (4)
N1—C1—C2—Cl1	2.5 (3)	Cl2—C11—C12—C13	179.7 (2)
C1—C2—C3—C4	−0.7 (3)	C11—C12—C13—C14	−0.5 (4)
Cl1—C2—C3—C4	179.50 (18)	C12—C13—C14—C15	−0.3 (4)
C2—C3—C4—C5	0.3 (4)	C11—C10—C15—C14	−0.8 (4)
C3—C4—C5—C6	0.0 (4)	N2—C10—C15—C14	179.5 (2)
C4—C5—C6—C1	0.0 (4)	C13—C14—C15—C10	1.0 (4)
C2—C1—C6—C5	−0.4 (3)	O1—C7—N1—C1	−2.9 (3)
N1—C1—C6—C5	177.61 (19)	C8—C7—N1—C1	176.46 (18)
O1—C7—C8—C9	101.8 (2)	C6—C1—N1—C7	26.0 (3)
N1—C7—C8—C9	−77.5 (2)	C2—C1—N1—C7	−156.1 (2)
C7—C8—C9—O2	37.4 (3)	O2—C9—N2—C10	6.1 (3)
C7—C8—C9—N2	−145.36 (18)	C8—C9—N2—C10	−171.12 (19)
C15—C10—C11—C12	0.0 (4)	C15—C10—N2—C9	−43.0 (3)
N2—C10—C11—C12	179.7 (2)	C11—C10—N2—C9	137.2 (2)

### Hydrogen-bond geometry (Å, °)

**Table:**

Cg2 is the centroid of the C10/C15 phenyl ring.

**Table:**

*D*—H···*A*	*D*—H	H···*A*	*D*···*A*	*D*—H···*A*
N1—H1N···O2^i^	0.86	2.24	3.038 (2)	154
N2—H2N···O1^ii^	0.86	2.03	2.856 (2)	160
C8—H8A···O2^i^	0.97	2.43	3.219 (3)	138
C15—H15···O2^iii^	0.93	2.54	3.265 (3)	135
C3—H3···Cg2^iv^	0.93	2.74	3.608 (2)	155
C6—H6···O1	0.93	2.37	2.906 (3)	116
C15—H15···O2	0.93	2.52	2.916 (3)	106
N1—H1N···Cl1	0.86	2.58	2.9730 (18)	109

Symmetry codes: (i) *x*, −*y*+3/2, *z*+1/2; (ii) −*x*, −*y*+1, −*z*+1; (iii) *x*, −*y*+3/2, *z*−1/2; (iv) −*x*, *y*+1/2, −*z*+1/2.
